# Is it deontologically correct to promote your medical services? An ethical approach on medical marketing

**Published:** 2018

**Authors:** Consuela Mădălina Gheorghe

**Affiliations:** * Philologist, Authorized translator

Taking into account today’s modern and rapid development, marketing and mass media have become key elements of the organizational growth even in the healthcare field. This is because people are becoming more informed [ **1**] and have started looking for the answers to their medical problems and health-related information in the online environment [ **2**]. More and more studies report that an increasingly high percent of people would rather use the internet than see a doctor face-to-face regarding their medical problems. They feel more confident discussing with other people (peers, colleagues, relatives, or even strangers) online than with professionals. Unfortunately, professional opinions are a last resort when nothing else works. This may also be the reason why people want to be involved in their care and social media offers the empowerment they are looking for, suggesting that he is able to take control of his healthcare needs.

Web and mobile technologies are increasingly used in promoting and advertising healthcare services and, directly, support patient-centered care. Patients benefit from disease self-management tools, contact to others, and closer monitoring. Researchers study drug efficiency, or recruit patients for clinical studies via these technologies. However, in practice, low communication barriers in social-media, limited privacy and security issues lead to ethical issues [ **3**].

The literature on ethical issues emphasize that the aspects that require careful attention are confidence and privacy. In other words, physicians need to ensure that they keep the borders between private and professional intact, and, at the same time, preserve the patient’s anonymity when citing Internet content [ **3**] .

However, an online visible presence of both doctors and organizations needs to be developed in order to survive on the dynamic healthcare market. 

One method of making the healthcare organization and services visible is on the CRM platforms, which offer general exposure and allow patients and doctors to find information on top pages on Google and other search engines. 

Still, the most efficient ways in which healthcare services can be promoted are online and offline media. The online media include search engine optimized (SEO) blogs, websites, social media networks (Twitter, Facebook, YouTube), online forums, discussion boards, cost-effective paid advertising (PPC Campaigns), targeted email marketing campaigns, mobile marketing, video marketing. Studies have highlighted that many people select their healthcare provider based on the social media reputation [ **4**]. The offline media include TV, print, outdoor media, magazines, newspapers, etc., being as effective as online media in finding healthcare information.

When using the advertising media of healthcare services, it is also important to tackle the deontological and ethical aspects of it. Are there moral values and principles to be taken into account? Obviously, before going further, what should be mentioned is that a clear distinction should be made between “law” and “ethics”. Moreover, regarding the managerial ethics in healthcare, what should be taken into account when dealing with the primary sources of external ethical expectations concerning a hospital, are its stakeholders (patients, professionals, healthcare financing organizations, professional associations, the community in which the hospitals resides, etc.) [ **5**].

So, according to Merriam-Webster dictionary, ethics is defined as “a set of moral principles: a theory or system of moral values” [ **6**].

At the same time, Bioethics is the discipline that deals with the ethical implications of biological research and applications especially in medicine [ **7**]. It also tries to define the medical activity and other related activity needed to maintain the functioning of a health institution, through the development of moral values and, at the same time, combines various disciplines such as medicine, philosophy, law, sociology, and theology [ **8**]. At the same time, advertising and promotion are part of the strategy, whose aim is to maintain relationships with the target audience. To regulate the activity of advertising and promotion, ethical rules had to be established for healthcare marketing. Thus, it is important that promotional messages say and show the truth and do not create unjustified expectations. The services in the advertisement should be provided exactly as they are presented and marketing communications should be more consistent with reality, because the target public of these advertisements encompass people who are sick, and, in their desire to get well, are very vulnerable and try to buy any service that seems to comply with their desire to improve their health state, even if that particular service is not good or suitable for their illness. In addition, the presented medical service should not alter the reality and should not give false hopes to patients [ **8**].

In conclusion, a reflection of roles and responsibilities should be seriously taken into consideration when dealing with medical social-media in healthcare environment. Further, it is important that the data should not be used abusively and privacy and confidentiality of online and offline users of medical advertising should be preserved at all times. 

**Assist. Prof. Consuela-Mădălina Gheorghe, PhD,** **Philologist, Authorized translator**
